# Challenges to Changing the Culture of Parenting in Japan

**DOI:** 10.2188/jea.JE20190265

**Published:** 2020-10-05

**Authors:** Aya Goto, Pamela J. Surkan, Michael R. Reich

**Affiliations:** 1 Center for Integrated Science and Humanities, Fukushima Medical University, Fukushima, Japan; 2 Department of International Health, Johns Hopkins Bloomberg School of Health, Baltimore, MD, USA; 3 Department of Global Health and Population, Harvard T.H. Chan School of Public Health, Boston, MA, USA

Japanese society suffers from an odd paradox related to children: Japan is simultaneously experiencing a declining number of live births and a rising number of child abuse cases. Between 2005 and 2015, the number of live births decreased from 1,062,530 to 1,005,677, while during the same period the number of child abuse cases rose from 34,472 to 103,286.^1^ During the decade, the incidence rate of reported child abuse cases for 0–2 years old per 1,000 children of the same age showed a steep increase from 2 to 7. While children in Japan are considered invaluable for the development of society, they are at an increasing risk of harsh or in some cases life threatening parenting. Regarding this issue, Baba and colleagues report that, even as the rate of spanking was declining, over 60% of 3-year-old toddlers born in 2010 were sometimes or always spanked by family members.^2^ The ninth edition (2017) of Dr. Benjamin Spock’s book on baby care, the United States’ famous guide for parents first published in 1945, states, “In the olden days, most children were spanked on the assumption that this was necessary to make them behave. In the twenty-first century, … [parents] have come to realize that children can be well behaved, cooperative, and polite without ever having been punished physically”.^3^ This statement reflects a major change of parenting culture in the United States.

Japan is going through a similar transition, but at a rather slow pace. An international comparison of parenting published in 2001 notes that Japanese maternal and child health professionals were less strict than British and American professionals about preventing corporal punishment.^4^ The study reviewed child care books from the 1980s and found that Japanese professionals even mentioned a need of and a way of spanking to be used when children behaved in ways that could possibly hurt themselves. More recently, as Baba and colleagues describe in their study background, the 2019 amendments of the “Act on the Prevention, etc of Child Abuse” in Japan included a ban on corporal punishment of children by parents and other guardians, to go into effect in April 2020. Changing the law alone, however, is not enough to change people’s behaviors. Recognizing this limitation, the Ministry of Health, Labour and Welfare started a campaign in 2019 to ban the “Whip of Love” (“*Ai no Muchi*” is a popular term used in Japanese to refer to violent discipline or corporal punishment of children).^5^ But this awareness-raising campaign will need the support of other interventions to build parents’ capacity in using non-violent forms of discipline in Japan.^6^

Prevention of violence against children is included in Goal 16 of the United Nations’ Sustainable Development Goals. As of September 2019, 57 countries had national laws that prohibited all corporal punishment of children.^7^ Interventions to support the law can occur on many levels, ranging from indicated initiatives (targeting parents who maltreat), selective efforts (targeting parents generally and those who work with children), and universal interventions (mass education).^8^ While many indicated and selective initiatives have been implemented, there has been a dearth of rigorous evaluations of these programs.^8^ In addition, the effects of interventions on parenting or social norms around spanking are rarely studied, which ultimately could be important for making change.^9^

The characteristics described in Baba et al’s article that were associated with corporal punishment reflect important features of current Japanese society. According to the article, being a boy, living with other siblings, having younger parents, and coming from a household with lower socioeconomic status were risk factors for being spanked. On the other hand, if the child was living with both parents or grandparents and the child’s father was the survey respondent, the risk was lower. These results indicate three important issues to address: traditional parenting styles, social disparities, and the need for fathers’ involvement in parenting.

The first and third issues are connected. An in-depth study on parenting among Japanese two-sibling families pointed to strong birth order and gender effects on children’s perceived parental style.^10^ The elder male children reported paternal parenting style as more rejecting, which was explained by traditional Japanese fathers’ expectations that their elder sons be a family role model. Even in Western culture, a study from Netherlands reported that fathers with strong stereotypical gender-role attitudes used more parenting strategies employing physical control with boys than with girls.^11^ Parenting style is transferred from one generation to the next to some extent.^12^ At the same time, it is culturally and socially patterned. One study among Japanese fathers living in Hawaii reported how they altered their traditional authoritarian gender role to a more family-oriented style under the influence of Western culture.^13^ Making paternal parenting a norm and providing equal opportunities for fathers to receive the same parenting support as mothers will hopefully lead to gender-equality in parenting in the next generation.

The second issue related to social disparities and positive parenting also involves intergenerational transmission. Similar to Baba et al’s findings, another article from the *Journal of Epidemiology* found economic adversity was associated with either shaking or smothering among parents of 4-month-old infants.^14^ Child abuse is disproportionately prevalent among people with low socioeconomic status,^15^ and the experience of childhood physical discipline is associated with subsequent use of physical discipline as parents.^16^ There is a need to stop the intergenerational transmission of negative parenting among socially disadvantaged groups.

In relation to social disparities, Baba and colleagues explained the association between spanking and unstable work mainly being attributable to fewer work hours and longer time spent at home. One recent article in the *Journal of Epidemiology* reported limited access to center-based childhood education among socioeconomically disadvantaged children in Japan, although the childcare fee was set according to household income to reduce economic barriers to enrollment.^17^ More pro-active measures are required to encourage effective use of childcare facilities by disadvantaged families. In response, the Japanese government is starting a new program in October 2019, offering free preschool education and childcare for low-income families with children up to 2 years old and all families with children of 3 to 5 years old. The effects of this program should be closely monitored.

As illustrated in Baba et al’s article, more social and political efforts are needed for “changing the culture of parenting” in Japan, especially among men and among socially disadvantaged groups. Prime Minister Shinzo Abe’s “Womenomics” aims to create a society in which “all women can shine.” He seeks to empower women to participate and take a lead socially, economically, and politically, mainly by increasing the availability of nursing care as stated above, childcare leave benefits, and reforming work culture. We (along with many others) are skeptical that these measures (mostly supply-side efforts) will be effective in changing the culture of parenting in Japan. Rather than building gender-specific strategies based on the male-breadwinner model, Japan needs social change strategies that are gender-equal and social disparity-focused, and that address the deep cultural roots of parenting styles.

Achieving such changes in parenting culture requires efforts not only in rewriting organizational policy, but also in assuring actual implementation. Brinton and Mun interviewed Japanese human resource managers about implementation of parental leave, and found that some managers implicitly assumed that family policies pertain only to female employees.^18^ They concluded that the answer to Japanese women’s low participation in workforce “lies not only in the content of Japan’s family policies but in managers’ interpretation of policy purposes in the context of labour market structure and the prevailing cultural model of household gender relations.” Male employees also need to be confident that taking off time for parental leave will not result in negative consequences for their advancement at work.

In conclusion, changing the culture of parenting in Japan requires concerted and creative multilevel efforts to move beyond rhetoric to actions. These actions should include evaluations of how current family support policies are functioning in Japan and should focus interventions on changing the dynamics of family units rather than reinforcing gender-specific strategies. If women are encouraged to shine in society, men should also be encouraged to shine at home. By having women and men share responsibilities at work and at home, family policies will become more effective and Japan’s odd paradox of increased child abuse (including corporal punishment) directed at fewer babies will hopefully decline.
